# Evaluation of Trilysine-Cross-Linked Gellan Gum for Intratumoral Delivery of Anti-PD‑1 in a Colorectal Cancer Mouse Tumor Model

**DOI:** 10.1021/acsbiomaterials.5c01503

**Published:** 2026-01-23

**Authors:** Carolina Villarreal-Otalvaro, Francini Luna, Rosangel A. Ramos Espinoza, Eric D. Lombardini, Zephyr Paxton, Luis Vidali, Jeannine M. Coburn

**Affiliations:** † Department of Biomedical Engineering, 8718 Worcester Polytechnic Institute, Worcester, Massachusetts 01609, United States; ‡ 5724 Boston Scientific Corporation, Marlborough, Massachusetts 01752, United States; § Department of Biology and Biotechnology, Worcester Polytechnic Institute, Worcester, Massachusetts 01609, United States; ∥ Bioinformatics and Computational Biology Program, Worcester Polytechnic Institute, Worcester, Massachusetts 01609, United States

**Keywords:** aPD-1 release, hydrogel, intratumoral delivery, systemic injection, colorectal cancer

## Abstract

Colorectal cancer (CRC) is the second leading cause of death in the United States in the adult population. When detected at early stages, the survival rate is higher than 70% but when diagnosed at a metastatic stage, the 5-year survival rate significantly declines to 15%. In recent years, immunotherapy has shown promising results in a selective patient population with advanced or metastatic CRC with microsatellite instability (MSI) and mismatch repair (MMR). Hydrogels are a growing field of research to improve the delivery of monoclonal antibodies. In this work, a gellan gum-based (GG) hydrogel noncovalently cross-linked using trilysine (TLA) was characterized for its drug release and diffusion properties. A partial release of the checkpoint inhibitor anti-programmed death-1 (aPD-1) was observed with an almost 40% cumulative release within the first 24 h. FRAP analysis showed varying diffusion rates (μm^2^/s) for Atto 488-IgG (2.63 ± 0.32), 60–76 kDa FITC-Dextran (2.65 ± 0.31), and 150 kDa FITC-Dextran (2.80 ± 0.83), with a 70–90% recovery postbleaching. Additionally, intratumoral delivery of aPD-1 was evaluated in a CRC mouse (C57BL/6) tumor model inoculated with MC38 cells. Quantification of aPD-1 in the plasma and tumor showed an increase in concentration by 1.5- and 3-fold, respectively, when delivered using the intratumoral hydrogel route in comparison with either intraperitoneal (i.p.) or drug-free intratumoral administration. Overall, this noncytotoxic hydrogel provided an alternative delivery route for aPD-1, maximizing its presence both in circulating blood and in the treatment site, serving as a simple localized delivery system in CRC.

## Graphical abstract

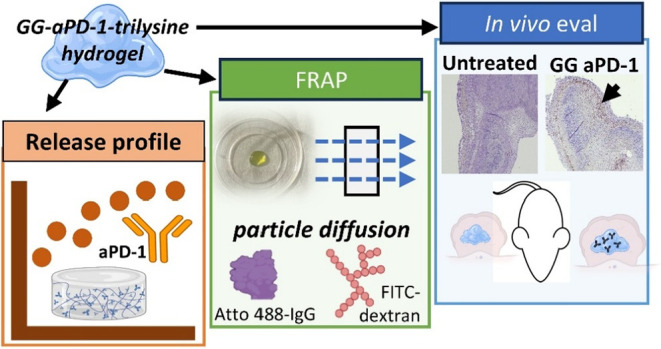

## Introduction

Colorectal cancer (CRC) is a significant health concern, being the third most commonly diagnosed and the second leading cause of cancer death in the United States in the adult population.^1^ Its incidence among men and women younger than 55 years increased 2% in 2024, making it the fourth leading cause of death in this patient population.^2^ CRC is the abnormal growth of tumor cells in the large intestine and rectum and staging varies based on the tumor, node, and metastasis classification.^3^ The survival rate for locoregional CRC (I–III stages) is 70–90%, but when detected at advanced-stage metastasis (stage IV), it drops to 13–17%.^4,5^ It is highly clinical and pathologically heterogeneous, and current treatment options include surgical resection, chemotherapy, radiotherapy, targeted therapy, and immunotherapy.^6,7^ Immunotherapy, particularly checkpoint inhibitors such as aPD-1, has shown promise as a treatment option by targeting the programmed death-1 (PD-1) receptor on T cells. This enhances the immune system’s ability to fight cancer by blocking the interaction with the programmed death ligand-1 (PD-L1) receptor present on the malignant cells.^7^ In 2017, the Food and Drug Administration (FDA) approved two aPD-1 treatments (pembrolizumab and nivolumab) for patients with metastatic microsatellite instability-high (MSI-high), refractory MSI-high, mismatch repair (MMR) metastatic, and unresectable CRC that showed an objective response rate of 28%.^8^ MSI is the result of germline mutations such as insertions or deletions involved in the DNA MMR repair pathway; and MMR is a mechanism that repairs errors in the DNA.^5,9^

The effectiveness of these treatments can be limited by systemic side effects such as kidney failure, colitis, enterocolitis, endocrine dysfunction, autoimmune reaction,^5,10–13^ and poor drug delivery to the tumor site. Additionally, overcoming challenging biological barriers, such as the tumor microenvironment, remains an obstacle that can hinder therapeutic efficacy.^14^ The development of localized delivery systems represents a promising strategy to mitigate systemic side effects and address biological barriers effectively. For instance, Si et al. developed an 8-arm polyethylene glycol (PEG) amine and an oxidized dextran implantable biopolymer to deliver oxaliplatin (chemotherapy) and resiquimod (immune adjuvant) for the treatment of peritoneal metastasis carcinoma.^15^ Wang et al. developed pH-responsive nanoparticles made of 2-methacryloyloxyethyl phosphorylcholine and PEG methacrylate to deliver aPD-L1 for the treatment of glioblastomas.^16^ Li et al. used an alginate hydrogel system for intratumoral delivery of aPD-1 and celecoxib in a melanoma mice tumor model.^17^ Kinetic analysis demonstrated high levels of the antibody in the serum for up to 2 weeks, resulting in tumor size reduction and demonstrating intratumoral therapy retention, bringing potential benefits such as reduced toxicity, cost, concentration, and drug administration frequency.^17^ These data are an indication of the possibility of local delivery of aPD-1 as an alternative route to systemic delivery when using injectable hydrogels.

Localized delivery of cancer therapeutics can increase therapeutic efficacy while minimizing systemic toxicity.^18,19^ This approach involves delivering drugs directly to the tumor site in varying form factors, including nanoparticles, patches, wafers, and hydrogels,^6,20–22^ ensuring higher local concentrations and reducing adverse effects on healthy tissues. Researchers have explored GG as a delivery vehicle of anti-inflammatory compounds, antibiotics, or anticancer drugs for bladder, prostate, breast, and nonsmall cell lung cancer applications and oral cancer.^23–29^ Anti-CD3/CD28 antibody-conjugated gellan gum (GG) nanoparticles have demonstrated the ability to serve as a T cell activator downregulating a cytotoxic response.^30^ Additional drug performance with combined therapies (paclitaxel and prednisolone) or as a postsurgical treatment has resulted in improved antitumor activity;^25^ however, its use with checkpoint inhibitors, such as aPD-1, is yet to be explored. Prior work with TLA-cross-linked GG hydrogels elucidated the ability to deliver IgG as an analogue for monoclonal antibodies, with varying cumulative mass released at day five, ranging from ∼40 to 79% at different loading concentrations.^31,32^

In our prior work, we characterized TLA-GG hydrogels formulated by varying TLA and GG concentrations (0.5 and 1.0%), optimizing formulations that supported homogeneous hydrogel formation, and subsequently evaluated the mechanical properties via rheological and injection force characterization. When varying TLA (0.01–0.05%), there was a concentration-dependent increase in injection force and elastic-like properties, though at 0.01% TLA, a more fluid-like behavior was observed. All injection force values were found to be within an acceptable range for injectable biomaterials.^33^ Additionally, we reported the sustained release of immunoglobulin G and *in vitro* cytocompatibility using normal human dermal fibroblasts.

The aim of this work is to present a potential neoadjuvant or adjuvant therapy for intratumoral delivery of aPD-1 via TLA cross-linking of GG hydrogels, to be used in combination with standard practices for patients who are candidates to receive immunotherapy. Here, we evaluated the release profile of aPD-1 with different drug loading contents via *in vitro* testing. Due to known retention challenges of the antibody within the matrix, the diffusivity of molecules of varying shapes and molecular weights through a GG hydrogel via FRAP analysis was characterized. Finally, the intratumoral delivery of aPD-1 via the developed hydrogel was evaluated in a CRC murine model and compared to systemic delivery, represented via intraperitoneal (i.p.) injections. Findings suggest that intratumoral delivery of aPD-1 can help in avoiding cytotoxic side effects, given the off-target nature of systemic delivery while maximizing the concentration of the treatment agent intratumorally.

## Materials and Methods

### Materials

Gellan gum low acyl was provided by CP Kelco. Trilysine acetate was procured from Bachem, CA. Ultrapure water was obtained from a PicoPure water purification system (Hydro Service and Supplies, Durham, NC). Syringes (Beckton Dickinson), syringe caps (Air-Tite), syringe filters (0.22 μm, Foxx Life Sciences), low-binding microcentrifuge tubes, bovine serum albumin (BSA), DPBS Ca^2+^/Mg^2+^ (Quality Biological), treated 96-well plates with a flat bottom (Nunc-Immuno, Thermo Fisher Scientific), 2 N sulfuric acid (stop solution), human IgG (lyophilized, fractionated, and purified) (Innovate Research), and the BioFX TMB extended-range HRP microwell substrate (Surmodics) were obtained from VWR (Radnor, PA). aPD-1 (InVivoMAb antimouse PD-1 (CD279)) and aPD-1 dilution buffer (InVivoPure pH 7.0 dilution buffer) were obtained from BioXcell (Lebanon, NH). Mouse PD-1 was obtained from ACROBiosystems (Newark, DE). Peroxidase AffiniPure Goat Anti-Rat IgG, Fcγ frag specific (HRP peroxidase), was purchased from Jackson ImmunoResearch (West Grove, PA). The MC38 cell line was obtained from Kerafast (Boston, MA). DMEM with a high glucose concentration was purchased from Cytiva (Marlborough, MA). Fetal bovine serum (FBS), nonessential ammino acids (10 mM stock), sodium pyruvate (100 mM), glutamine (200 mM stock), gentamicin (50 mg/mL stock), HEPES buffer (1 M stock), penicillin–streptomycin (10,000 units/mL penicillin and 10,000 μg/mL streptomycin stock), and protease inhibitor mini tablets were purchased from Thermo Fisher Scientific (Waltham, MA). The glass bottom dish was obtained from Mattek (Ashland, MA). FITC-Dextran (average molecular weight of 60,000–76 000 g/mol), FITC-Dextran (average molecular weight of 150 000 g/mol (FD150S)), and Atto 488 protein labeling kit were procured from Sigma-Aldrich. The sterile saline solution was purchased from Quality Biological (Gaithersburg, MD).

### aPD-1 Hydrogel Fabrication

Hydrogels were prepared as previously reported with some modification.^31^ Briefly, a bubble-free solution of 1% GG in ultrapure water was obtained after constant agitation at 70 °C, then the temperature was lowered to 38 °C, and 800 μL was transferred to a 1 mL syringe. Separately, 100 μL of 0.4% TLA and aPD-1 (1 mg/mL or 500 μg/mL) solutions were preloaded individually in 1 mL syringes. GG and aPD-1 were mixed back and forth with both syringes coupled with a female luer-to-luer connector, forming a GG + aPD-1 solution. Finally, the TLA solution was syringe-mixed with the GG + aPD-1 solution. Hydrogels were stored at 4 °C for about 24 h prior to testing. For sterile preparations, all solutions and hydrogel handling were performed inside a biosafety cabinet. The same method was employed with all individual solutions filtered and sterilized (0.22 μm) for hydrogels used in *in vivo* evaluations.

### Physical Characterization of Hydrogels

To understand the potential impact that the antibody could have on the hydrogel, physical characterization with varying concentrations of aPD-1 was performed through injection force and rheology testing. GG hydrogels were prepared as previously described with varying concentrations of aPD-1 (0, 5, and 10 μg) and characterized by injection force and rheology. Antibody dilution buffer (BioXcell, InVivoPure) was used for dissolving aPD-1 at the respective concentration.

Injection force was determined using an Instron machine via axial force (N) from three replicas, with a 50 N load cell at a rate of 2 mm/min. Viscosity (η*Pa.s), storage modulus (*G*′), and loss modulus (*G*″) evaluations were performed using a DHR-1 rheometer (60 mm cone plate, 1° angle, 37 °C) at a frequency of 1–100 Hz from three replicas.

### *In Vitro* aPD-1 Release

Drug delivery of thermosensitive molecules such as the monoclonal IgG antibody, using a TLA-cross-linked GG hydrogel system, has been reported previously.^31,32^ In this work, IgG was replaced with the immune checkpoint inhibitor, aPD-1, and aPD-1 release was evaluated for up to 5 days with two IgG loading amounts (5 and 10 μg/hydrogel). Hydrogel samples (∼100 mg per sample) were incubated in release media (PBS) at 37 °C. The release media were sampled at predetermined time points (900 μL; 1 h–28 d) and replaced with fresh PBS (900 μL).

### aPD-1 Quantification via ELISA

Quantification of aPD-1 was determined via an indirect enzyme-linked immunosorbent assay (ELISA), with modifications to previously reported protocols (Figure S1).^17,34^ The output of this ELISA protocol reflects the affinity and binding specificity between aPD-1 and PD-1, enabling the accurate measurement of the antibody response. This demonstrates that the antibody remains stable and active.^17,35–37^ ELISA plates were coated overnight for 16 h at 4 °C with 1 μg/mL mouse recombinant PD-1 protein and protected from light. Content was removed via decantation and blocked with a 5% BSA solution in DPBS Ca^2+^/Mg^2+^ for 1 h at 37 °C. Wells were washed 4 times with 0.05% Tween-20 in PBS. The same aPD-1 was used as the test sample and standard curve. For the standard curve, the antibody was first diluted to an initial concentration of 200 μg/mL using InVivoPure pH 7.0 dilution buffer. Subsequently, a serial dilution (1:1) was performed using 1% BSA in PBS. Then, 100 μL of the sample, or the standard solution, was transferred to the precoated plate and incubated for 1 h at 37 °C followed by washing 4× with 0.05% Tween-20 in PBS. For detection, the wells were loaded with 0.25 μg/mL HRP peroxidase affiniPure Goat Anti-Rat IgG, Fcγ fragment specific in 1% BSA dilution buffer solution, and incubated for 1 h at 37 °C followed by washing 4× with 0.05% Tween-20 in PBS. The TMB substrate solution (100 μL) was added to the wells and incubated for ∼10 min at room temperature followed by the addition of 2 N sulfuric acid stop solution. The absorbance was measured using a plate reader (SpectraMax M2 multilabel microplate reader; Molecular Devices Inc., Santa Clara, CA) at 450 nm. The final concentration was determined based on the standard curve values (200–0.003 μg/mL) using a 4-parameter logistic regression.

### Diffusion Characterization via FRAP Analysis

Diffusion characterization through the GG hydrogel was evaluated via FRAP analysis. Hydrogels (10 mL final volume) were prepared as previously reported with some modification.^31^ Once a bubble-free solution of 1% GG solution in ultrapure water was obtained, the temperature was lowered to 38 °C. Then, 1 mL of 0.2 mg/mL FITC-Dextran or conjugated IgG solution was added under constant agitation at 38 °C for ∼1 min at 700 rpm. The TLA cross-linker was added and mixed for another ∼1 min. The contents of the solutions were immediately transferred to a 100 mm Petri dish and allowed to set. Samples were parafilm-sealed, protected from light with a tin foil, and stored at 4 °C for at least 24 h prior to testing. IgG was conjugated with Atto 488 using a commercially available Atto 488 protein labeling kit, following the manufacturer’s instructions for sample preparation and analysis.

A hydrogel sample (Ø, 6 mm; H, ∼1–1.5 mm) was obtained using a biopsy punch and transferred to a confocal compliant glass bottom dish. Three separate samples were evaluated using a Leica Stellaris 8 confocal microscope. A circular region of interest (ROI) of 15 μm in diameter was defined, bleached for 15 s (laser: 100% power, 488 nm), with 180 s recovery, using a zoom factor of 4, at a speed of 600 Hz with a 20× lens. Raw data obtained from Leica was processed using the Excel Analyzer feature for the curve fit:

1 $$f\left(t\right)=A\left(1-e^{-\tau t}\right)$$

where *f*(*t*) is the fluorescence intensity normalized to the prebleach fluorescence intensity, *A* is the mobile fraction, and τ is the exponential fit constant. From τ, the half-life (τ_1/2_) and estimated diffusion coefficients (*D*) were derived.

2 $$\tau_{1/2}=\frac{\ln\left(0.5\right)}{-\tau }$$

3 $$D=\frac{0.88\times w^{2}}{\left(4\times \tau_{1/2}\right)}$$

where *w* is the bleach radius (7.5 μm). Data was normalized to the respective prebleach results. Additional normalization to account for the fluorescence immediately after photobleaching not being equal to zero was calculated as reported by Day and Kang.^38^

4 $$\mathrm{Mobile fraction}=\frac{F_{\infty }-F_{0}}{F_{i}-F_{0}}$$

where *F*_*∞*_ is the fluorescence intensity as time goes to infinity, *F*_i_ is the prebleached fluorescence intensity, and *F*_0_ is the fluorescence intensity immediately after photobleaching.

### Maintenance of the MC38 Cell Line

The colorectal tumor cell MC38 cell line was cultured as instructed by the vendor in DMEM supplemented with 10% FBS, nonessential amino acids (0.1 mM), sodium pyruvate (1 mM), glutamine (2 mM), gentamicin sulfate (50 μg/mL), HEPES buffer (1 mM), 100 units/mL penicillin, and 100 μg/mL streptomycin stock. Cells were maintained in a 5% CO_2_ atmosphere at 37 °C and passaged via trypsinization when they reached 80% confluence.

### MC38 Cytocompatibility

Cell viability was assessed using the MC38 cell line through indirect metabolic activity via a resazurin assay using a modified method previously reported.^31,39,40^ MC38 cells were incubated for 48 h in growth media in a 24-well plate (2.5 × 10^3^ or 5 × 10^3^ cells per well) with a sterilized hydrogel and a control (no hydrogel), followed by performing the resazurin assay (3 h; 37.5 °C). The fluorescence signal was measured with a SpectraMax M2 multilabel microplate reader (Molecular Devices Inc., CA). Data was normalized to untreated cells.

### Mouse Subcutaneous Colorectal Cancer Model, Tumor Volume Monitoring, and Treatment

All mouse procedures were performed in compliance with Worcester Polytechnic Institute’s guidelines for appropriate care and use of animals and were maintained and handled under protocols approved by the Institutional Animal Care and Use (protocol #21-130). A suspension of 10^6^ MC38 cells in a 50 μL saline solution was injected (25 Ga, 1/2 mL syringe) into the flank area of female mice C57BL/6 (6–8 weeks old, Charles River, MA). The overall behavior and weight of animals were monitored. Mice were euthanized when abnormal trends in weight or ulceration were noticed. Tumor growth was monitored at least 3 times per week with the neoplasms being measured using calipers, and the tumor volume was determined by the equation below:

5 $$Tumor\;volume\left({mm}^{3}\right):\frac{xy^{2}}{2}$$

where the length is represented by *x* and *y*, with *y* being the shortest length taken across the narrowest part of the tumor. Once tumors reached ∼200–250 mm^3^, intervention (“treatment day”) was performed per assigned experimental group (*n* = 5, each). *Experimental group:* intratumoral delivery of aPD-1 loaded in the GG hydrogel (GG aPD-1); *drug treatment controls:* intratumoral aPD-1 free drug (intratumoral aPD-1), intraperitoneal aPD-1 (i.p. aPD-1) free drug; and *no drug treatment controls:* untreated and intratumoral delivery of GG only. Animals were assigned to two cohorts: 3-day or terminal. Mice were euthanized 3 days post treatment and when the tumor volume exceeded ∼1500 mm^3^ (*terminal*). Tumors were evaluated histologically and via immunohistochemistry. Additionally, the plasma was collected and plasma- and tumor-associated aPD-1 was quantified (Scheme 1).

**Scheme 1:**
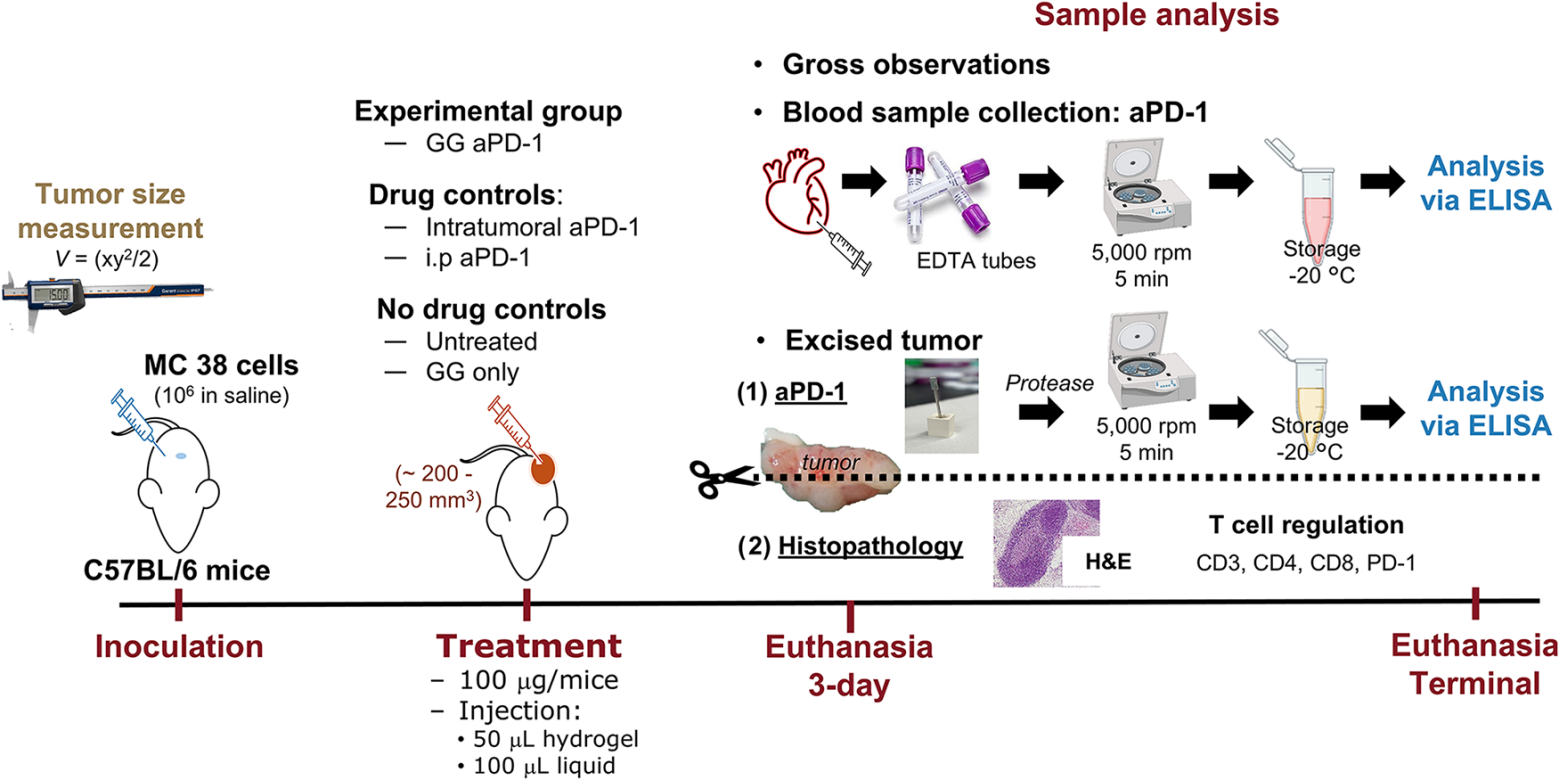
Visual Representation of CRC *In Vivo* Study^a^

a Evaluation of tumor growth of MC38 cancer cells injected subcutaneously on the flank of female C57BL/6 mice (6–8 weeks old). Mice were subjected to systemic and intratumoral delivery of aPD-1 with and without the GG-based hydrogel or remained untreated once tumors reached 200–250 mm^3^.

### Histopathology and Immunohistochemistry

Tumors harvested from the mice from the 3-day or terminal cohorts (postmortem) were weighed and cut in half. One half was fixed in 10% formalin for 48 h, rinsed with PBS, and stored in 70% ethanol at 4 °C before being transferred to Applied Pathology Systems (Shrewsbury, MA). After a series of ethanol gradient dehydration through xylene, the tissues were embedded in paraffin and sectioned at 5 μm for histochemical staining and immunohistochemistry staining.

Hematoxylin and eosin (H&E) staining was performed in an automated stainer (Leica Autostainer XL) following vendor’s internal procedures. H&E slides were blindly reviewed by a board-certified veterinary pathologist who provided qualitative impressions and scoring. A semiqualitative evaluation was used based on the scoring matrix system (Table 1).

**Table 1:** H&E Scoring Matrix

Score	Inflammation
0	Within normal limits
1	Minimal; benign features to the neoplasm; minimal expansion or replacement of preexisting architecture; low mitotic rate (∼1–3/high power field (HPF)); rare single-cell necrosis
2	Mild; neoplastic expansion with a predominantly uniform phenotype of neoplastic cells and mitotic rate (<6/HPF); single-cell necrosis; mild numbers of inflammatory cells
3	Moderate; neoplastic invasion with cytomegaly, karyomegaly, and a high mitotic rate (<12/HPF) with rare, bizarre mitotic figures; tumoral necrosis and single-cell necrosis; mild numbers of inflammatory cells; rare peritheliomatous survival of neoplastic cells
4	Marked; significant neoplastic invasion with cytomegaly, karyomegaly, and a high mitotic rate (12–15/HPF) with occasional bizarre mitotic figures; tumoral necrosis and destruction of local architecture; mild to moderate numbers of inflammatory cells; occasional peritheliomatous survival of neoplastic cells
5	Severe; overwhelming neoplastic invasion with extreme cytomegaly, karyomegaly, and a high mitotic rate (>15/HPF) with bizarre mitotic figures; large regions of necrosis and destruction of local architecture; moderate to high numbers of inflammatory cells; peritheliomatous survival of neoplastic cells

Immunohistochemistry staining for CD3, CD4, CD8, and PD-1 was performed using a commercial detection kit (Vector Laboratories, MP-7601) with a Dako autostainer. Paraffin sections were dewaxed, rehydrated, and subjected to heat-induced epitope retrieval (HIER) in Tris-based buffer. Slides were blocked with BLOXALL blocking buffer and 2.5% horse serum prior to 1 h incubation with CD3, CD4, CD8, and PD-1 antibody at 1:200, 1:4000, 1:1000, and 1:100 dilution, respectively (CD3 Abcam, # ab135372; CD4: Abcam, # ab183685; CD8: CST, #98941; PD-1: CST, # 84651). Subsequently, the sections were incubated with antirabbit amplifier antibody and ImmPRESS Excel polymer reagent sequentially before incubation with DAB chromogen. The slides were then counterstained with hematoxylin, followed by dehydration and coverslipping. Immunohistochemistry-stained sections followed a qualitative analysis based on the presence and distribution of positively stained CD3, CD4, CD8, and PD-1 cells.

### Tumor-Associated and Circulating aPD-1 Quantitation

The second half of the harvested tumor was weighed and homogenized with protease inhibitor mini tablets diluted in PBS using a mortar and pestle. The solution was centrifuged for 5 min at 5000 rpm (Onilab, Walnut, CA) and the supernatant was stored at −20 °C for tumor-associated aPD-1 quantitation.

For aPD-1 quantification in plasma, blood was rapidly obtained following necropsy via cardiac puncture, agglutinated in EDTA tubes, and centrifuged for 5 min at 5000 rpm. The plasma was collected and stored at −20 °C prior to testing. The aPD-1 concentration was determined via ELISA as described in Section 4.2.3. The *Estimated total aPD-1 in plasma* (μg) was calculated using the aPD-1 concentration, the mouse weight at EOL (g), and the estimated total blood volume in mouse (0.095 mL/g).^41^ The *Estimated total aPD-1 in total tumor* (μg) was calculated using the aPD-1 concentration in half tumor (μg/g tumor) and full tumor weight (g).

### Statistical Analysis

Data analysis was conducted with the statistical software Minitab. Normality and variance were evaluated for all data sets. A one-way analysis of variance (ANOVA) with a significant difference of *p* < 0.05, followed by a Tukey’s HSD pairwise comparison, was performed for multiple group comparison.

## Results and Discussion

### Physical Characterization of GG Hydrogels

GG is an anionic polysaccharide that is typically cross-linked with mono- or divalent ions. The hydrogel under evaluation forms through ionic interaction between the amines of TLA and the carboxylate residues of glucuronic acid, as well as hydrogen bonding.^31^ aPD-1 is incorporated using a calcium- and magnesium-free solution, ensuring that the physical characteristics of the hydrogel remain unchanged. The injection force was not impacted by the incorporation of aPD-1 with no statistically significant difference among groups and comparable values of 15 N when using a 1 mL syringe and a 22 Ga needle (Figure S2). Additionally, rheological results showed a similar solidlike behavior (Figure S3A) and a similar complex viscosity (Figure S3B) for most of the frequency sweep of 1–100 Hz.

### aPD-1 *In Vitro* Release

Localized delivery of therapeutic agents is a growing field of clinical research, given its potential to spare healthy cells and minimize cytotoxicity to unwanted organs.^18,42^ In this work, the release profile of aPD-1 from GG hydrogels was investigated with varying amounts of aPD-1 loading (5 or 10 μg per hydrogel). Despite extended-release evaluation for 28 d (Figure S4), a partial release of aPD-1 from GG hydrogels was achieved, with a maximum cumulative release of 46.2 ± 32.2 and 34.5 ± 9.4%, for 10 μg of aPD-1 and 5 μg of aPD-1 loading per hydrogel, respectively (Figure 1). A similar release profile in PBS (pH 7.4) was observed with the model molecule, IgG, with a 41.9 ± 22.5% elution,^31^ suggesting that aPD-1 and IgG share comparable release characteristics due to their similar properties.

**Figure 1:**
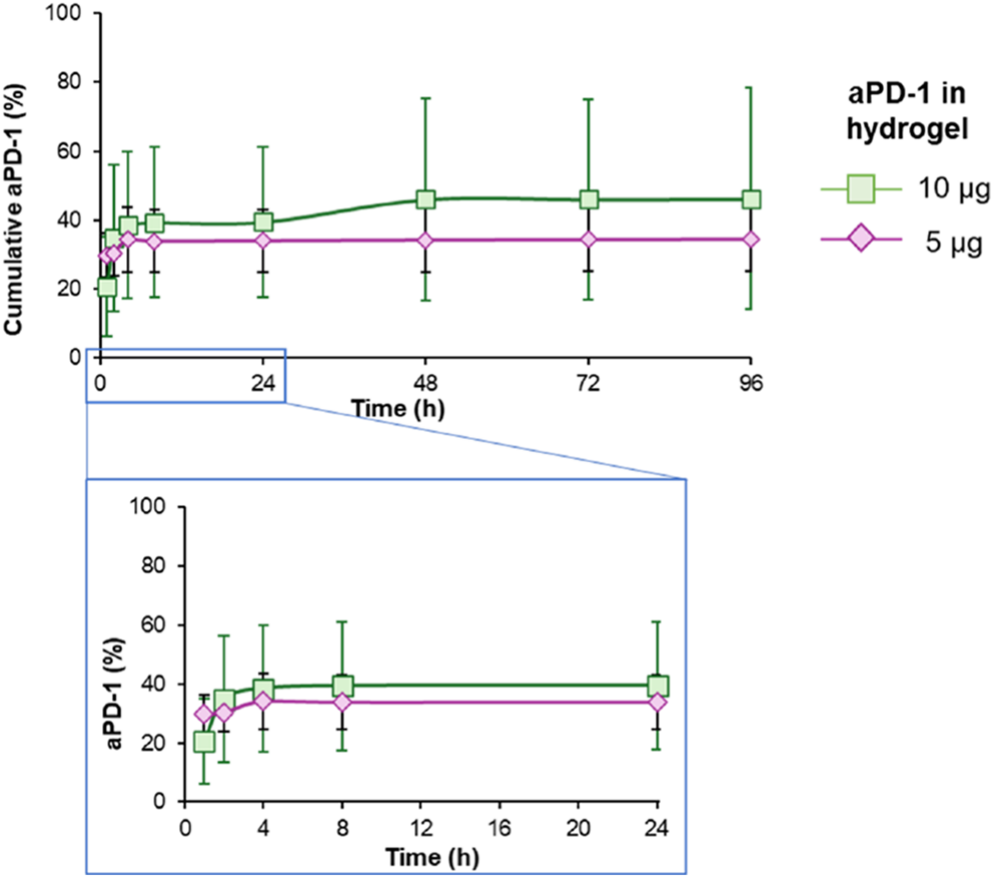
Cumulative release of aPD-1 from GG hydrogels. Samples were loaded with 10 and 5 μg of aPD-1. Representative data are shown from three independent samples. Magnified release at 24 h is shown in the bottom panel.

Differences in mass released between 5 and 10 μg aPD-1 per hydrogel could be associated with inherent variability with the fabrication process via the syringe mixing method, which is reflected by the large standard deviation and could represent a challenge for mass production. Nonetheless, a system with a higher loading of the active drug was chosen for *in vivo* evaluations to maximize the potential therapeutic effect. Alternative processing techniques should be further explored to reduce the variation, maximize delivery, extend release duration, and scale-up manufacturing. In our prior work, we utilized a 10 mL-scale mixing system within a heated block, which was feasible due to the low cost of prototypical immunoglobulin.^31^ However, this scale was cost-prohibitive for this work studying aPD-1. For translational development, a larger-scale formulation and development would be warranted. An example approach could be the use of mechanical agitation in a temperature-controlled environment utilizing insulated jackets equipped with thermocouples to monitor and manage temperature fluctuations.^43–45^ Another example is managing transitions between processing steps, such as shifting from mixing to syringe filling, to maintain at a temperature above the gelation point.^46,47^ Additionally, carriers responsive to environmental stimuli (i.e., reactive species or pH) are among some of the successful strategies reported by others to improve the release amounts of antibodies.^16,48,49^ For example, Chen et al. investigated an albumin-based complex, cross-linked with a reactive oxygen species (ROS)-sensitive cross-linker, in a melanoma cancer model for the release of antiintegrin-associated protein (aCD47) and aPD-1. The ROS-responsive hydrogel resulted in a higher release of aPD-1 (%) at approximately 24 h in PBS with H_2_O_2_ as compared to when it was absent (∼70 vs ∼10%, respectively). This suggests that release media and the interaction with the ROS-sensitive cross-linker influenced a higher aPD-1 release,^48^ as compared to our GG hydrogel. Wang et al. reported pH-responsive smart nanoparticles designed to overcome the blood-brain barrier, where approximately 60% cumulative release of IgG was achieved within 24 h in a release media at pH 6.0, compared to ∼20% at pH 7.4.^16^ Finally, Wang et al. developed *in situ* self-assembly nanotubes conjugated to iRGD, a hydrophilic peptide able to penetrate the tumor tissue due to binding to integrins on tumor and tumor-associated cells, for the codelivery of the chemotherapeutic agent, camptothecin, and aPD-1 in a brain cancer model. Direct conjugation of the drug to the peptide resulted in susceptibility to enzymatic degradation through metalloproteinase-2, with a cumulative drug release of ∼75% at 30 days.^49^

Reported extracellular pH values range from pH 6 to 7,^50^ which may impact drug release from ionic-cross-linked GG hydrogels. Others have reported that GG hydrogel compaction occurs as the pH dropped from 7.0 to 5.3 to 3.5 lower pH values.^51^ Zhang et al. reported the effect of pH on the properties of GG hydrogels cross-linked with Ca^2+^. The work showed that pH values of 3 and 4 resulted in stiffer, more elastic-like hydrogels than at pH values of 5 or higher, though there were slight pH-dependent differences between pH 5 and pH 7. These findings coincide with the p*K*_a_ of GG hydrogels, which has been reported to be around pH 3.5.^52^ Because of tumor-induced extracellular acidosis, the pH environment may differ from those studied for the *in vitro* release work reported here (pH 7.4 PBS). However, since the extracellular pH of tumors is far from where significant pH effects on GG hydrogel properties have been reported, it is not expected that relevant extracellular tumor pH conditions would drastically alter the release kinetics, though it warrants future investigation.

Furthermore, the ion concentration of the tumor microenvironment differs from that of the healthy tissue. It has been reported that a range of physiological relevant ions were found to have lower concentrations within colorectal tumors than nontumor colorectal tissue.^53^ Though several papers report *in vitro* drug release performed in PBS for applications in cancer treatment,^54–59^ it may be beneficial to fully characterize the impact of ion concentration and ionic composition on the release from GG hydrogels to develop a clearer understanding of how environmental conditions may impact protein release.

### Diffusion Characterization via FRAP Analysis

To characterize the molecular mobility within the hydrogels, the motility of molecules through the GG hydrogel was characterized via FRAP analysis using Atto 488-conjugated IgG (Atto 488-IgG) and FITC-Dextran (60–76 and 150 kDa). The GG hydrogels allowed for the diffusion of both Atto 488-IgG and FITC-Dextran molecules; however, complete recovery after the postbleached phase was not observed (Figure 2A). Using eq 1 to fit the normalized fluorescence intensity as a function of time allows for calculation of the apparent diffusion coefficient from τ, the half-life, and the bleach radius using eqs 2 and 3, respectively (Table 2). Among the conditions evaluated, Atto 488-IgG had the highest recovery, followed by 60–76 kDa FITC–Dextran and, finally, 150 kDa FITC-Dextran (Figure 2B,C). Atto 488-IgG showed the least resistance to diffusion with a mobile fraction of 0.94 ± 0.02. Lower-molecular-weight FITC-Dextran exhibited a faster recovery as compared to higher-molecular-weight FITC-Dextran with mobile fractions of 0.84 ± 0.01 and 0.73 ± 0.06, respectively (Table 2). Normalized data to the fluorescence intensity immediately after photobleaching using eq 4 confirmed these findings with similar recovery trends. Atto 488-IgG exhibited the least resistance to diffusion with a mobile fraction of 0.84 ± 0.04. Additionally, the fastest recovery was obtained with 60–76 kDa as compared to 150 kDa FITC-Dextran, with mobile fractions of 0.70 ± 0.06 and 0.43 ± 0.09, respectively. Such differences in the mobile fraction, particularly for Atto 488-IgG and 150 kDa FITC-Dextran, which have comparable molecular weights, are likely associated with the molecular structure and shape and potential interaction with the GG hydrogel. For instance, Atto 488-IgG is a globular protein, while FITC-Dextran is a highly branched polysaccharide commonly used to study the diffusivity of molecules in hydrogels.^60–62^ Similar results have also been reported in other hydrogel systems with varying cross-linking densities or FITC-Dextran molecular weights.^62–65^ These results show the prevalence of the immobile fraction, which is in agreement with the incomplete aPD-1 release data discussed above, due to potential entrapment in the bulk of the hydrogel.

**Figure 2:**
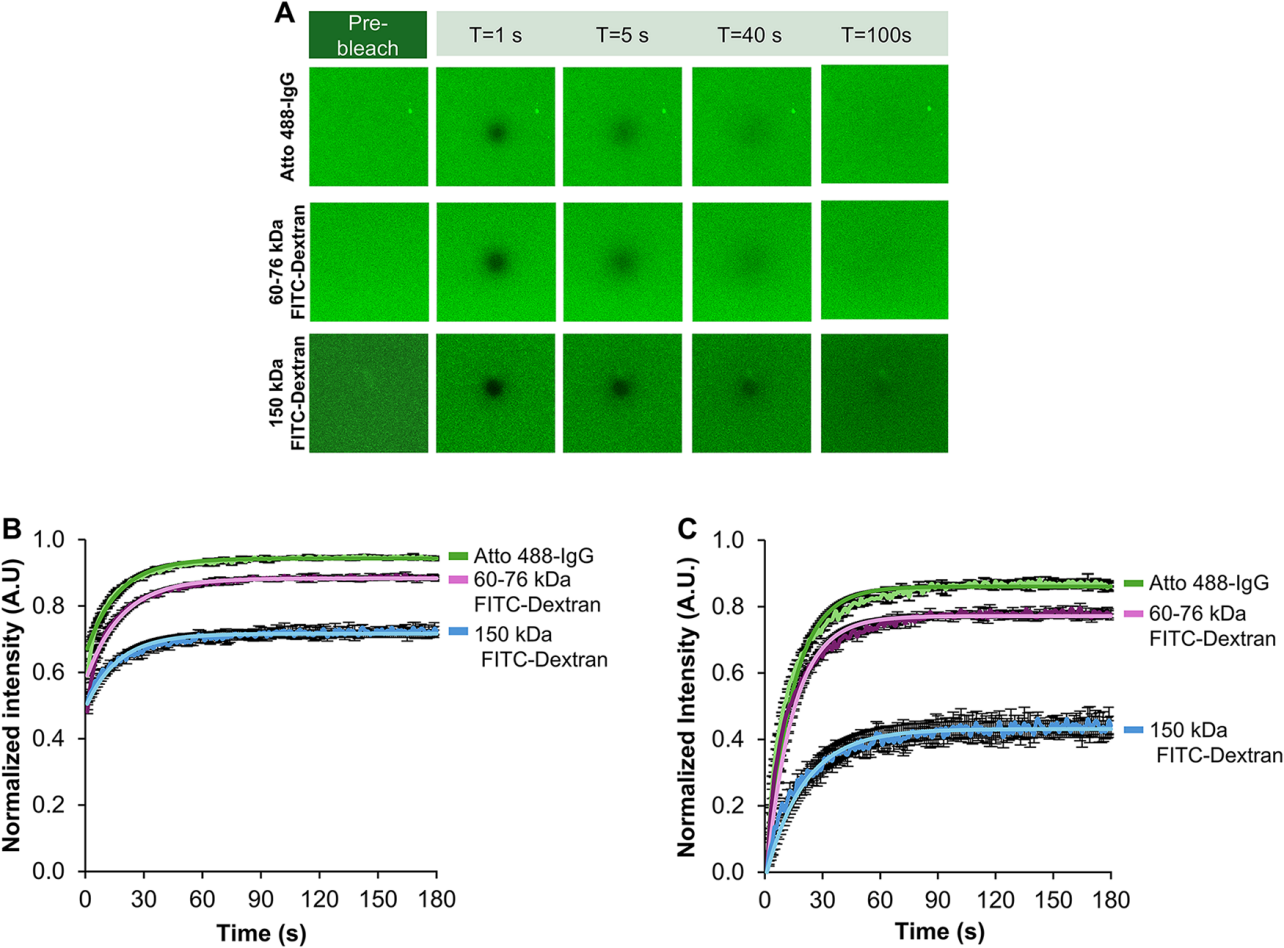
FRAP analysis of Atto 488-IgG and 60–76 and 150 kDa FITC-Dextran images. (A) Visual representation of the recovery of the photobleached region at varying time points (1–100 s). (B) Normalized intensity and (C) postbleached adjusted values of representative replicas from each group showed that Atto 488-IgG had the fastest recovery, while 150 kDa FITC-Dextran exhibited the slowest recovery. Data are presented as mean ± std deviation of three independent samples with three replicas.

**Table 2:** Diffusion Parameter Result of Atto 488-IgG and FITC-Dextran^a^

Data processing method	Parameters	Atto 488-IgG	60–76 kDa FITC-Dextran	150 kDa FITC-Dextran
$\frac{F_{t}}{F_{\mathrm{pre}-\mathrm{bleach}}}$	Mobile fraction	0.94 ± 0.02	0.84 ± 0.01	0.73 ± 0.06
	Immobile fraction	0.06 ± 0.02	0.13 ± 0.01	0.27 ± 0.06
	Diffusion constant, *D* (μm^2^/s)	2.63 ± 0.32	2.65 ± 0.31	2.80 ± 0.83
$\frac{F_{t}-F_{t=0,\mathrm{post}-\mathrm{bleach}}}{F_{\mathrm{pre}-\mathrm{bleach}-F_{t=0,\mathrm{post}-\mathrm{bleach}}}}$	Mobile fraction	0.84 ± 0.04	0.70 ± 0.06	0.43 ± 0.09
	Immobile fraction	0.16 ± 0.04	0.30 ± 0.06	0.57 ± 0.09
	Diffusion constant, *D* (μm^2^/s)	2.83 ± 0.53	3.53 ± 0.51	2.31 ± 0.45

a Definitions of each term in the equations: *F_t_* = fluorescence intensity at each time point; *F*_pre‑bleach_ = average prebleached fluorescence intensity; *F_t_*_=0_,_post‑bleach_ = fluorescence intensity immediately after sample bleaching.

The apparent diffusion coefficients were similar for all macromolecules evaluated. There are limitations to the experimental technique utilized. In the data acquired, 100% bleaching was never achieved, as indicated by the first data points of the normalized intensity being greater than 0. Our image rate was about 1 s, while diffusion was occurring on the order of milliseconds. The rapid diffusion of molecules into the bleached area during the bleaching process likely impacts the quality of the early-time-point fitting data resulting in similar diffusion coefficients despite differences in the molecular weights of the macromolecules evaluated. Additionally, we utilized the disk FRAP method, while a line FRAP method assesses more accurately a smaller and linear bleached region in three-dimensional samples with rapid diffusion dynamics,^66^ where further evaluation will be required.

### *In Vitro* Cytocompatibility of MC38 Cancer Cells

The cytocompatibility of the GG hydrogel was evaluated through *in vitro* testing. Colorectal cancer cells (MC38) were used with two cell densities (∼1.3 × 10^3^ or ∼2.6 x10^3^ cells/cm^2^). No changes in cell viability were observed, with outcomes above 97% (Figure 3A,B), indicating that GG hydrogels were not cytotoxic. These findings are consistent with previously reported results, where the same hydrogel was evaluated with Normal Human Dermal Fibroblasts, with viability above 90%.^31,67^

**Figure 3:**
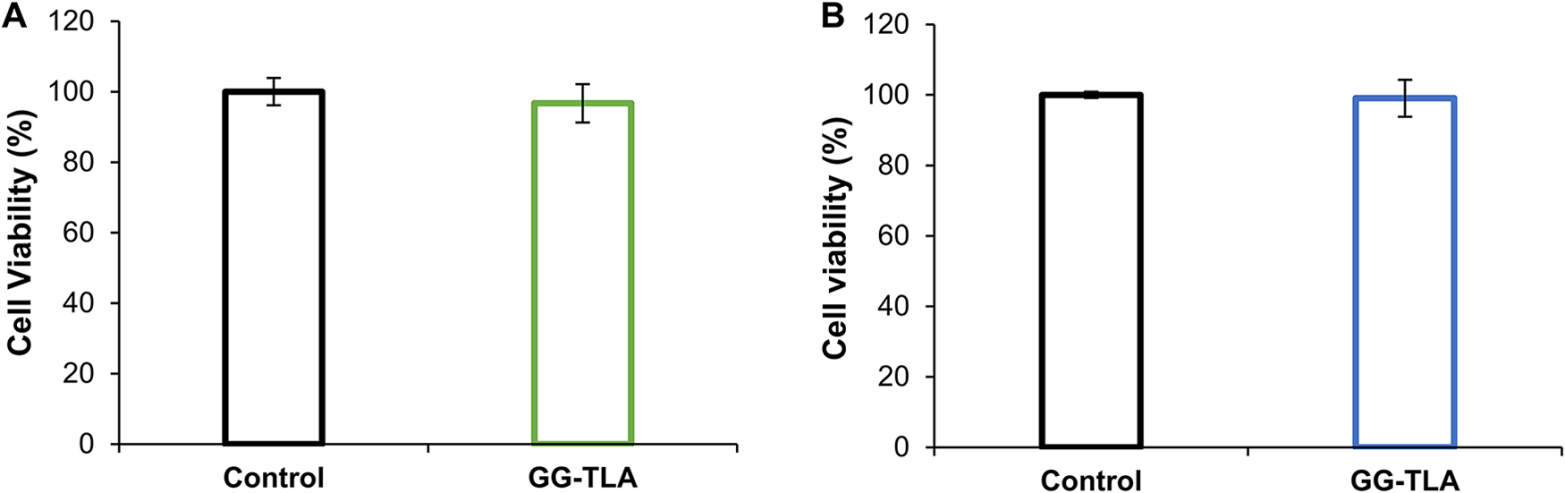
Viability of MC38 cells evaluated using a resazurin assay. Colorectal cancer cells at (A) 1300 and (B) 2600 cells/cm^2^ on a 24-well plate were exposed to a GG hydrogel (1% GG, 0.05% TLA), for 48 h at 37 °C. Data are presented as mean ± std deviation of three replicas; no significance differences (*p* > 0.05) among groups when compared to the control were found.

### *In Vivo* CRC Tumor Response

Subsequently, the efficacy of the combination therapy (GG aPD-1) was studied for intratumoral delivery in an *in vivo* CRC female C57BL6 mouse model. The study included the respective controls (untreated and GG only) and alternative treatment groups (intratumoral aPD-1, intraperitoneal). Upon intervention, the individual animal weight was monitored daily and reported normalized to the preintervention animal weight (Figure 4). Animals in the control groups exhibited minor variations in weight (Figure 4A,B). No changes in weight were observed in mice in groups where aPD-1 was supplied as a free drug intraperitoneal (Figure 4C, to represent systemic delivery) intratumorally (Figure 4D) or along with the hydrogel (Figure 4E). This suggests that the treatment resulted in negligible weight loss, suggesting that it was nontoxic and well tolerated.

**Figure 4:**
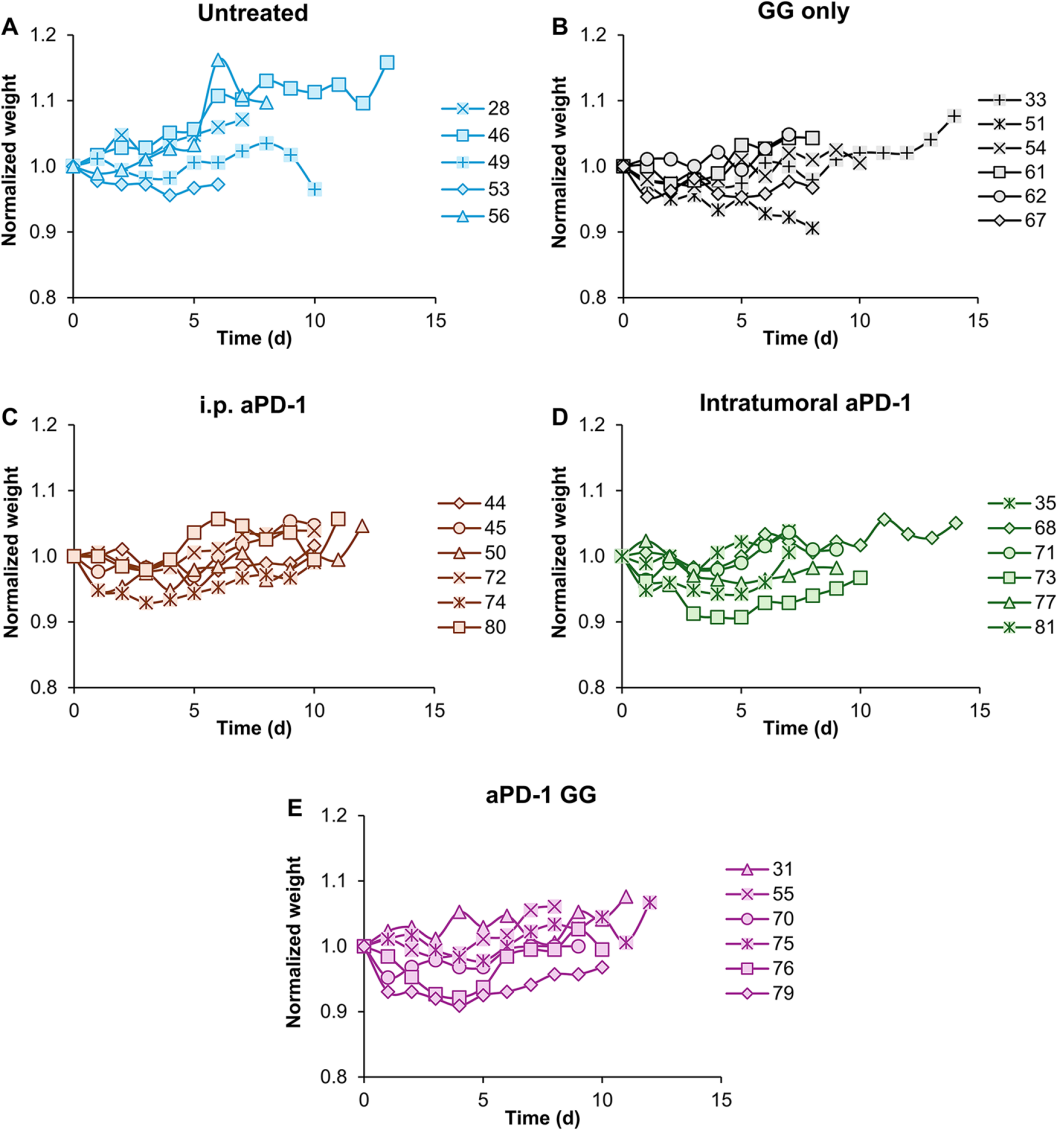
Changes in the weight of each mouse for the terminal cohort group. (A) Untreated (*n* = 5), (B) GG only (*n* = 6), (C) i.p. aPD-1 (*n* = 6), (D) intratumoral aPD-1 (*n* = 6), and (E) GG aPD-1 (*n* = 6). Data are presented as mean for each individual cohort group.

Individual tumor volumes were calculated as per eq 5 and individually monitored after intervention for all treatment groups and cohorts (Figure 5A–E). The time for the tumor volumes to reach 500, 1000, and 1500 mm^3^ was evaluated (Figure 5F). The untreated tumors and those receiving GG only (no drug treatment controls) reached an average volume of 500 mm^3^ in 5.4 ± 1.9 and 4.5 ± 2.2 days, respectively. For the i.p. aPD-1, intratumoral aPD-1, and GG aPD-1 groups, the tumors reached 500 mm^3^ in 6.0 ± 2.2, 6.5 ± 2.2, and 4.6 ± 1.9 days, respectively. Tumors reached an average volume of 1000 mm^3^ in 8.4 ± 2.4, 6.9 ± 2.4, 9.3 ± 1.1, 8.7 ± 2.7, and 8.4 ± 1.6 days for the untreated, GG only, i.p. aPD-1, intratumoral aPD-1, and GG aPD-1 groups, respectively.

**Figure 5:**
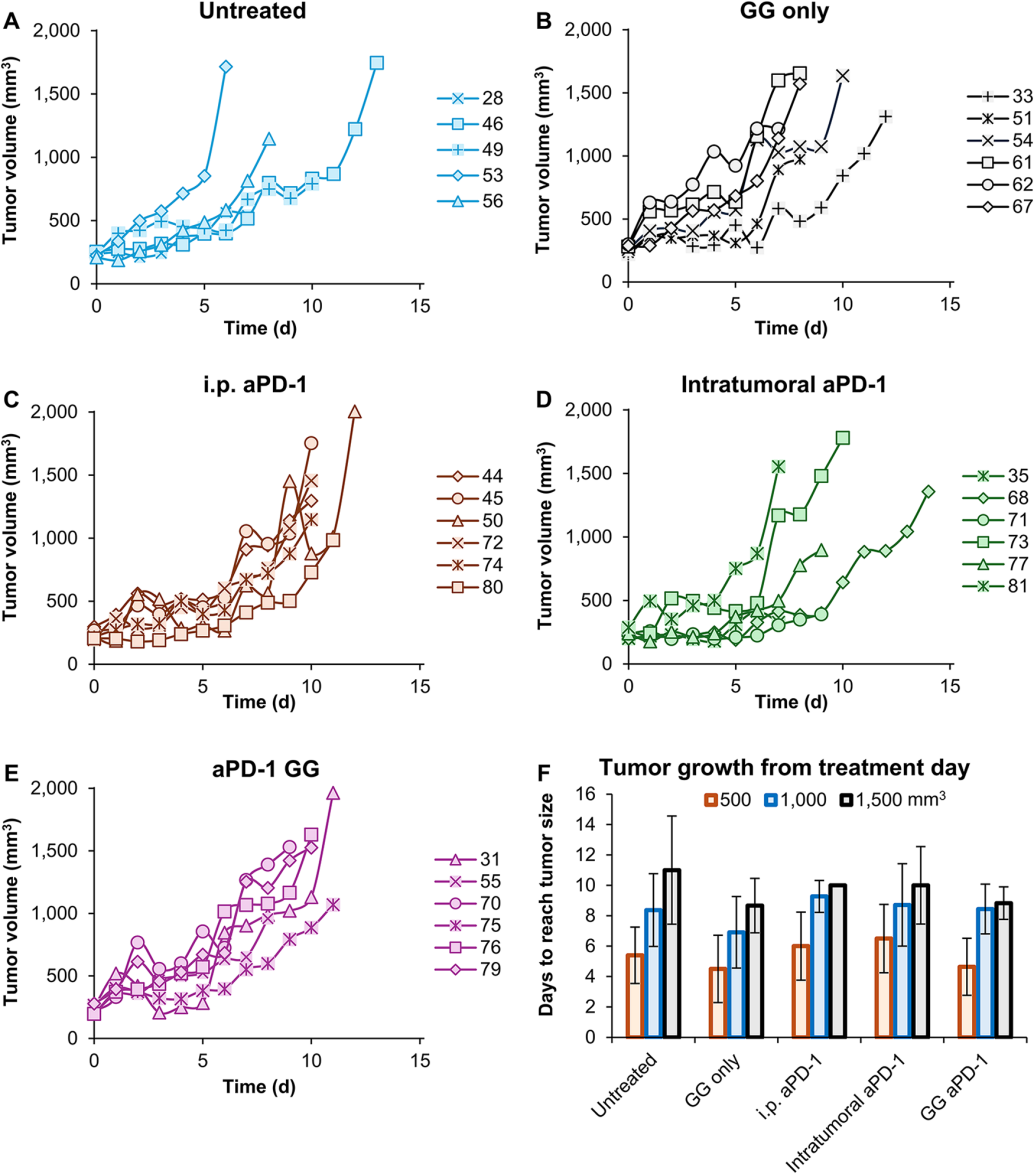
Tumor response to intervention. Tumor growth curves for the terminal cohort, with the time at which the tumors reach ∼200–250 mm^3^ adjusted to day = 0. (A) Untreated (*n* = 5), (B) GG only (*n* = 6), (C) i.p. aPD-1 (*n* = 6), (D) intratumoral aPD-1 (*n* = 6), and (E) GG aPD-1 (*n* = 6). Data are presented as mean (average number of animals for untreated *n* = 5; GG only *n* = 6; i.p. aPD-1 *n* = 6; intratumoral aPD-1 *n* = 6; and GG aPD-1 *n* = 6). (F) Number of days for MC38 tumors to reach 500, 1000 or 1500 mm^3^ and subjected to systemic and intratumoral delivery of aPD-1 with and without GG-based hydrogels or remained untreated after reaching intervention criteria of ∼200–250 mm^3^. Data are presented as mean ± std deviation. One-way Anova followed by Welch’s test showed no statistical significance among groups with a 95% confidence level and *p* < 0.05.

Finally, tumors reached an average volume of 1500 mm^3^ in 11.0 ± 3.6, 8.7 ± 1.8, 10.0, 10.0 ± 2.5, and 8.8 ± 1.1 days for the untreated, GG only, i.p. aPD-1, intratumoral aPD-1, and GG aPD-1 groups, respectively. Differences among the groups were not statistically significant, and superiority among the treatment groups with aPD-1 (free drug or with the hydrogel) was not observed, suggesting that a similar tumor response was achieved. Localized delivery of drugs is a growing field of research due to the advantages to minimize off-target treatment, increase focal retention, and potentially maximize the drug concentration on the treatment site.^68–70^ Therefore, additional *in vivo* characterization testing is needed to be further explored. For instance, intratumoral injections of aPD-1 (free drug or GG hydrogel) were performed without assisted visualization tools, such as ultrasound-guided or fluorescently tagged markers, which has enabled proper delivery, placement, and continuous drug distribution monitoring.^71–73^

### *In Vivo* Histopathological and Immunohistochemistry Outcomes

Histopathological and immunohistochemical (IHC) analysis was performed on the tumors collected at each time point (3-day and terminal). Hematoxylin and eosin (H&E) samples were evaluated using a semiqualitative scoring system based on general cell observations (Figure 6) and inflammation (Table 1).

**Figure 6:**
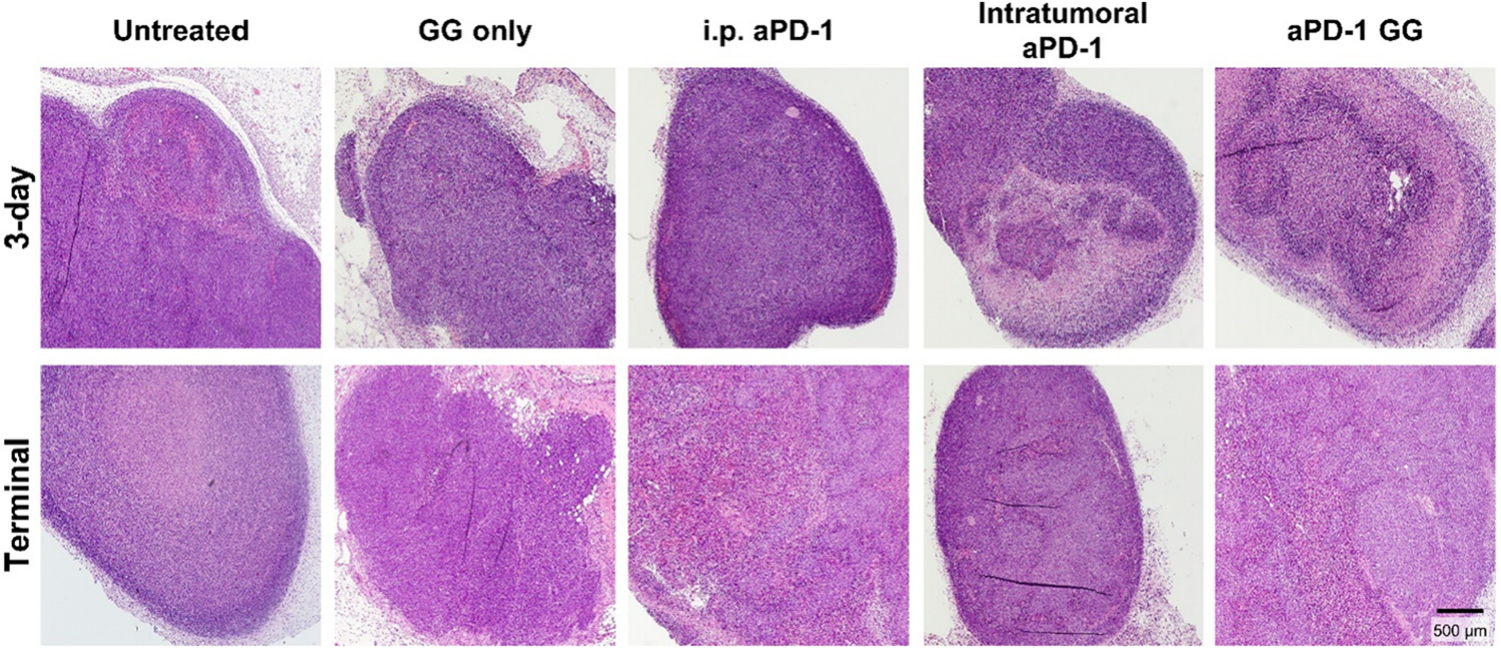
H&E images of representative samples from CRC tumor samples. The tumors were harvested post intervention at the 3-day time point (top row) and at the terminal time point (tumor volume >1500 mm^3^).

At day 3, the untreated and GG only-treated tumors presented with a well-demarcated, densely cellular neoplasm and contained necrotic regions, which is in alignment with observations reported by others.^74,75^ Overall, inflammation scores for the control and experimental groups ranged between mild and moderate (score of 2–3) (Figure 7), with dense streams, and disorganized islands of neoplastic cells, with peripheral inflammation. The i.p. aPD-1-treated tumors presented with neoplastic cells with irregular round to oval nuclei and moderate to marked amounts of granular amphophilic cytoplasm. The intratumoral aPD-1-treated tumors exhibited unencapsulated, well-demarcated, densely cellular, neoplastic cells with relatively distinct borders and moderate amounts of granular amphophilic cytoplasm with regional aggregates containing microvacuoles. The GG aPD-1-treated tumors showed disorganized streams and islands of neoplastic cells with a deep layer of palisading cells surrounding the necrotic cores, which could indicate a lack of oxygen.

**Figure 7:**
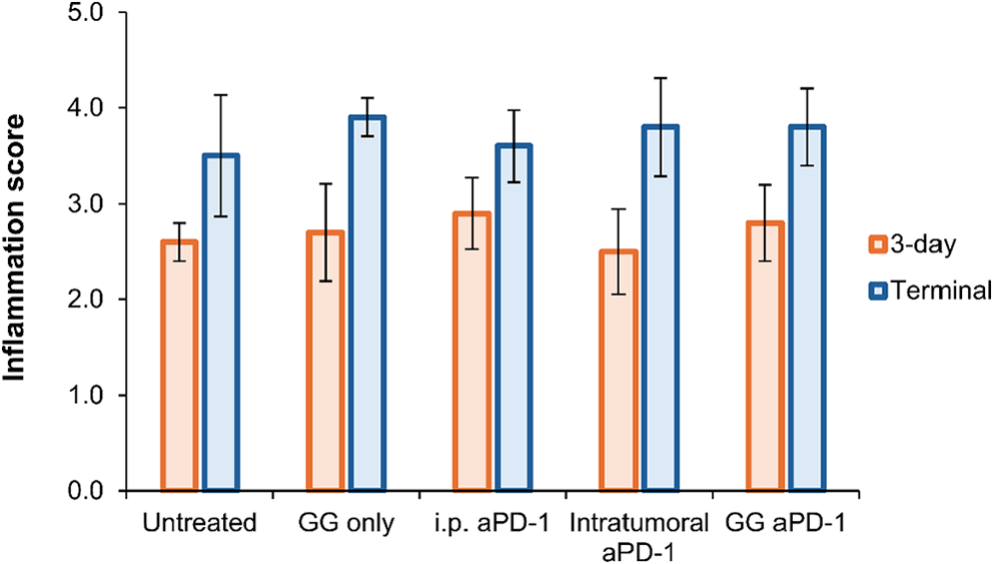
H&E inflammation of CRC tumor samples. Tumors harvested post intervention at the 3-day time point and at the terminal time point (tumor volume >1500 mm^3^) for untreated, GG only, i.p. aPD-1, intratumoral aPD-1, and GG aPD-1. Evaluations are based on inflammatory scores from Table 1. Data presented as mean ± standard deviation, *n* = 5. No statistically significant differences between groups and time points were found.

CRC is a very aggressive and rapidly growing tumor^76^ and given that no particular differences among treatment groups were observed via H&E staining, it suggests that interventions should have occurred at an earlier time point, when tumors were about 100 mm^3^ instead of 200–250 mm^3^, as reported by others.^74,77^ A smaller tumor volume may have favored a less immunosuppressive microenvironment and a more robust response to aPD-1 treatment.^78^ In the clinical setting, a large tumor burden is a metric known to be negatively correlated with immune checkpoint inhibitor efficacy.^79–83^ Furthermore, preclinical studies have reported improved outcomes with immune checkpoint inhibitors in lung squamous cell tumors and advanced ovarian tumors.^83,84^ At the terminal time point of this study, similarities among all treatment groups prevailed, including tumors with frequent single-cell necrosis and moderate numbers of lymphocytes, plasma cells, and rare neutrophils within the peripheral mucinous matrix. All tumors presented with effacing preexisting connective tissue intersected by large steams of necrosis with inflammatory scores of 4 (Figure 7). This supports our suspicion that intervention should have started at an earlier time point to better characterize cell population among treatment groups.^74^

IHC for CD3, CD4, CD8, and PD-1 was performed to characterize T cell infiltration and receptor expression due to their significance in CRC prognosis (Figure 8).^76^ The relevance of their characterization lies in the fact that the CD3 receptor triggers T cell activation and dictates the specificity of the immune response, and its presence in the intratumoral space may relate to the ability to respond to certain treatments.^85,86^ CD4-positive T cells, or helper T cells, stimulate killer T cells, macrophages, and B cells;^87^ and CD8-positive T cells stimulate immune response by recognizing and killing cancer cells.^88^ Qualitative analysis of the presence and distribution of positively stained cells, within or at the margins of the tumor, was conducted of all groups in both cohorts, 3-day and terminal. The untreated group showed low number-stained cells throughout the tumor positive for CD3, CD4, and CD8, at both 3-day and terminal time points. At the 3-day time point, the GG only group showed positively stained cells for CD3, CD4, and CD8 scattered through the tumor with higher prevalence in the margins at the terminal time point, which might be an indication of an immunosuppressed tumor environment. At the 3-day time point, i.p. aPD-1 treated tumors showed a lower presence of positively stained cells spread across the tumor for CD3, CD4, and CD8, but with a higher expression on the margins. However, at the terminal time point, a higher presence of positively stained cells for CD3, CD4, and CD8 was observed within the tumor, which might be an indication of an antitumor immune response tumor. Intratumoral aPD-1-treated tumors at the 3-day time point exhibited positively stained cells for CD3, CD4, and CD8, characterized by patchy clusters distributed within the tumor and reduced presence in the tumor margins. At the terminal time point, an increased presence of positively stained cells for CD3, CD4, and CD8 was observed relative to the 3-day time point. Finally, GG aPD-1-treated tumors at the 3-day time point showed positively stained cells for CD3, CD4, and CD8, distinguished by a diffuse distribution within the tumor and an elevated expression in the tumor margins. Similar to i.p. aPD-1-treated tumors, at the terminal time point, an increased presence of positively stained cells for CD3, CD4, and CD8 was observed within the tumor, potentially indicative of a tumor immune activated environment. Our results align with findings reported by Shields et al.^74^ Shields et al. examined the tumor T cell immune landscape to determine the optimal time point for preclinical studies with the MC38 cell line. Higher densities of CD3, CD4, and CD8 cells localized toward the outer tumor region with T cell exhaustion at later time points (14 and 21 days) were observed when inoculating 0.1 × 10^6^ MC38 cells.^74^ Similarities of T cell density among treatment groups may also be an indicator that future evaluations to better characterize T cell response when using GG for the localized intratumoral delivery of aPD-1 can be influenced by the cell density used during the inoculation phase. Evaluations with varying cell concentrations from 0.1 × 10^6^, 0.5 × 10^6^, and 1.0 × 10^6^ MC38 revealed a desirable tumor growth kinetics at 0.1 × 10^6^ cells, as compared to 1.0 × 10^6^ cells used in our study.

**Figure 8:**
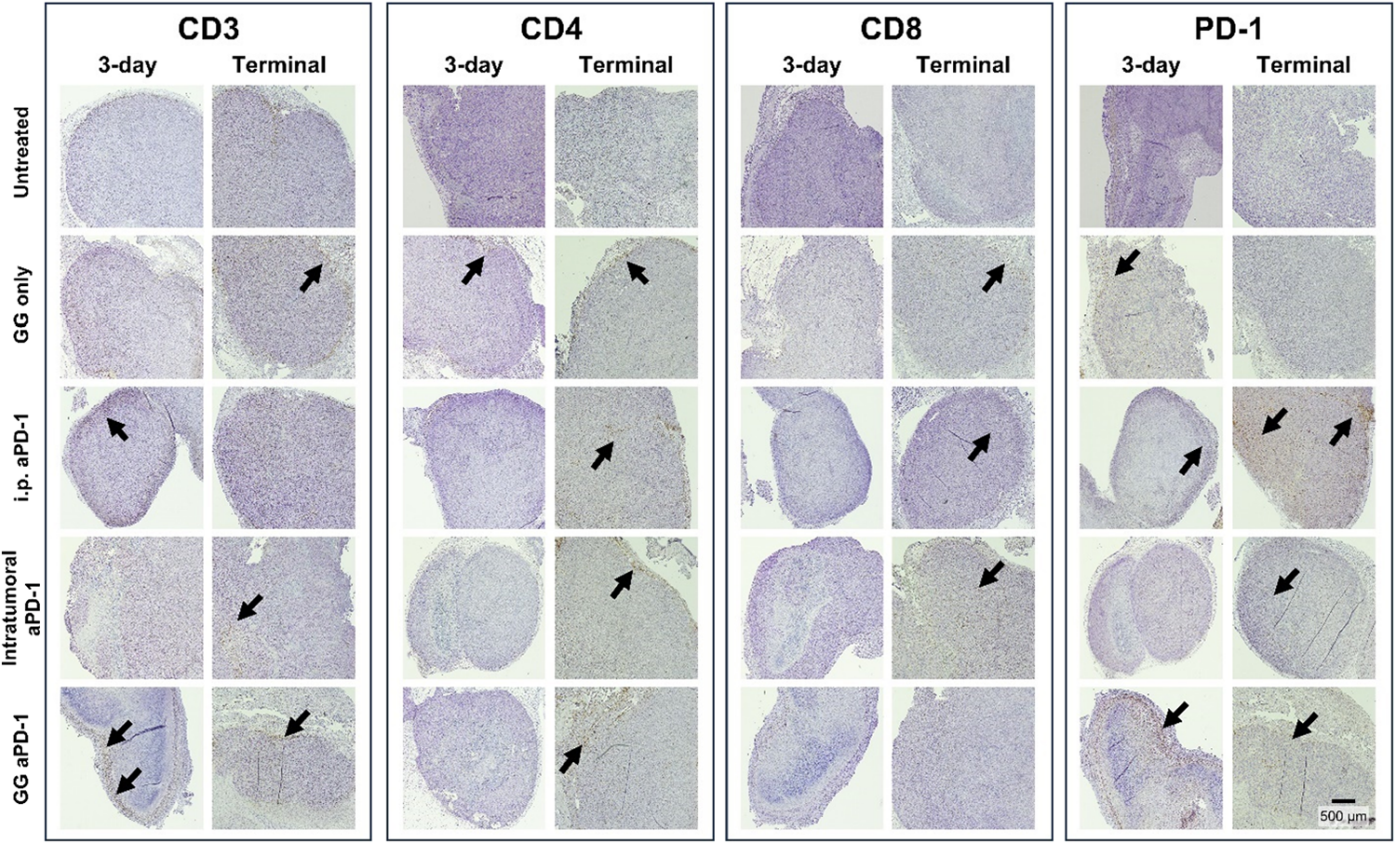
IHC images of representative CRC tumor samples. Tumors harvested post intervention at the 3-day time point and at the terminal time point (tumor volume >1500 mm^3^) for untreated, GG only, i.p. aPD-1, intratumoral aPD-1, and GG aPD-1. Arrows highlight positively stained cell markers present in the margins or within the tumor. Moderate CD3, CD4, CD8, and PD-1 observed in GG aPD-1 samples as compared to untreated followed by GG only, intratumoral aPD-1, and i.p. aPD-1. PD-1 marker prevalent at 3-day and terminal time points for i.p. aPD-1 and GG aPD-1.

As expected, the untreated tumors did not show any appreciable staining for PD-1 at either time point (Figure 8). At the 3-day time point, there was a higher presence of PD-1 in the GG aPD-1 treated tumors than in the i.p. aPD-1- and intratumoral aPD-1-treated tumor tissue, suggesting that the immunotherapy delivered via the hydrogel has a higher local concentration. Interestingly, PD-1-positive cells were scattered though the GG only-treated tumor, suggesting that the presence of foreign material could have promoted immune cell infiltration. At the terminal time point, there is a higher presence of PD-1-stained cells in the i.p. aPD-1- and GG aPD-1-treated tumors compared to intratumoral aPD-1-treated tumors. For i.p. aPD-1-treated tumors, the PD-1-positive cells were scattered through the tumor with clustering toward the margin, which might indicate a favorable immune cell activation. For intratumoral aPD-1-treated tumors, there was a lower presence of PD-1-positive cells both in tumor margins and throughout the tumor, potentially indicating immune cell depletion and suggesting the need for additional interventions. For GG aPD-1-treated tumors, a higher presence of PD-1-positively stained cells was mostly scattered through the tumor and margins, suggesting an antitumor immunity cell markup. The presence of immune cells scattered throughout the tumor represent an opportunity for combination therapy as well, particularly in CRC, where tumors are typically immunosuppressed.^77,89,90^ For instance, using chemotherapy or radiation therapy as a neoadjuvant agent to reduce tumor cells and minimize tumor size, followed by intratumoral delivery of immunotherapy, results in the creation of an immunoresponsive tumor microenvironment, as reported by others.^91–93^ Although qualitative IHC analysis revealed differences in the expression of CD3, CD4, CD8, and PD-1 among the different groups, the functional status of these tumor-infiltrating immune cells remains to be further characterized. Specifically, determining whether these cells are functional or exhausted, as reported by others,^78,94,95^ represents an important opportunity for further investigation.

### aPD-1 Quantitation in Tumors and Plasma

The GG aPD-1 hydrogel is presented herein as an alternative strategy approach to overcome the tumor barrier when delivering aPD-1 intratumorally. Harvested tumors and plasma at 3-day and terminal time points from all treatment groups were processed for aPD-1 quantification (Figure 9). Intratumoral aPD-1 quantification was performed to understand how aPD-1 is retained and its association to the delivery form (free drug, intraperitoneal, or with the hydrogel). In all treatment groups, aPD-1 was present at 3 days but negligible at the terminal (8–12 days post intervention). At 3 days, for i.p. aPD-1, intratumoral aPD-1, and GG aPD-1, the drug concentrations were 0.26 ± 0.29, 0.11 ± 0.06, and 0.40 ± 0.25 μg/mL, respectively, with a significant drop at the terminal (∼0.02 μg/mL for all groups) (Figure 9A). Circulating aPD-1 was calculated with plasma collected at EOL, which might represent a significant benefit for patients who suffer from metastatic disease. As expected, insignificant amounts of aPD-1 at 3-day and terminal levels were detected in the untreated (0.01 ± 0.001 μg/mL) and GG only (0.01 ± 0.001 μg/mL) control samples. The remaining groups had concentrations of aPD-1 of 0.10 ± 0.08, 0.11 ± 0.05, and 0.18 ± 0.09 μg/mL for i.p. aPD-1, intratumoral aPD-1, and GG aPD-1, respectively, with negligible presence at the terminal (Figure 9B).

**Figure 9:**
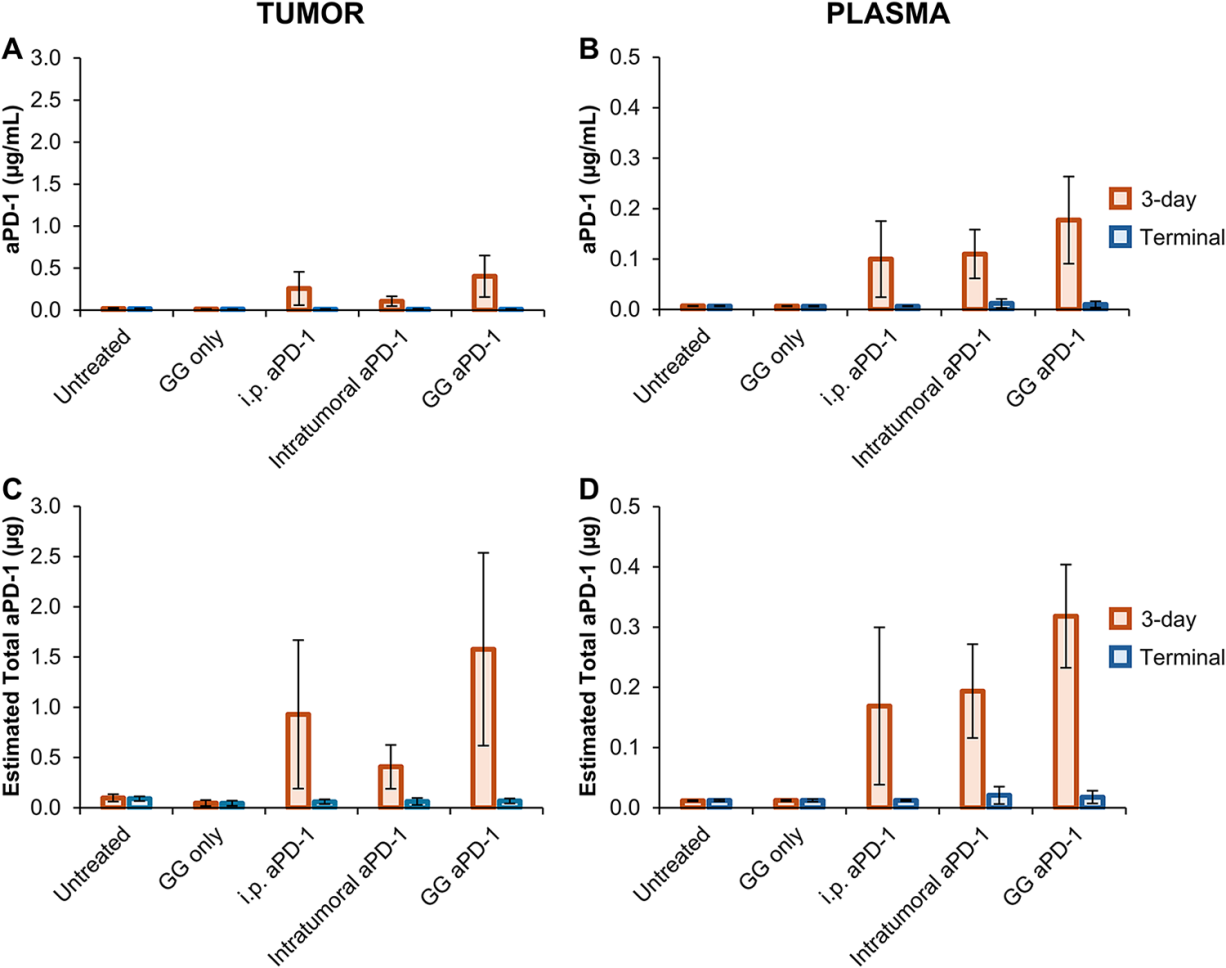
aPD-1 content in tumor and plasma. aPD-1 concentration in the (A) tumor and (B) plasma harvested post intervention at 3-day and terminal time points (tumor volume >1500 mm^3^). Estimated total aPD-1 in (C) tumors and (D) plasma. Results are presented as mean ± standard deviation with a minimum sample size of 3. The limited sample size for plasma groups is assignable to limited blood samples collected at the end of life.

Total aPD-1 (μg) in the tumor and plasma was estimated, considering the individual animal weight, tumor volume, and total blood volume reported in the literature.^41^ Results consistently indicated higher concentrations of aPD-1 in both tumor (Figure 9C) and plasma (Figure 9D) when delivered via the GG aPD-1 hydrogel system (tumor: 1.58 ± 0.96 μg; plasma 0.32 ± 0.09) as compared to i.p. (tumor: 0.93 ± 0.74 μg; plasma 0.17 ± 0.13 μg) or free drug intratumorally (tumor: 0.41 ±. 022 μg; plasma: 0.19 ± 0.08 μg). Our study highlights the significance of localized delivery to increase the drug concentration at the treatment site. This is in accordance with findings by Yamamoto et al. that reported an 8-fold increase in the intratumoral uptake of aPD-L1 when delivered via direct penetration to tumors in the intraperitoneal space.^96^

Li et al. found that an alginate-based hydrogel for aPD-1 delivery led to relatively high concentrations of aPD-1 serum in contrast to intraperitoneal administration.^17^ The hydrogel delivery also resulted in a 2-week presence of aPD-1 serum compared to a 1-week presence via intraperitoneal administration and a 1-day presence via subcutaneous injection of aPD-1 alone.^17^ At 3 days, we report similar trends where higher concentrations of circulating aPD-1 were found when delivered with the GG-based hydrogel, as compared to intraperitoneal or drug-free administration intratumorally.

Due to the predominant presence of inflammatory cells identified via H&E in the 3-day cohort for all treatment groups and the poor response to treatment, we speculate that treatment intervention should have started before the tumor reached 200–250 mm^3^ to better characterize the impact of aPD-1 with and without the intratumoral hydrogel delivery. For instance, Wang et al. performed local delivery of the hydrogel loaded with aPD-1 and camptothecin (chemotherapy) in a colon cancer model with CT 26 cells when tumors reached 100–150 mm^3^, resulting in 100% tumor regression.^49^ However, injection time, accurate intratumoral injection (i.e., assisted delivery via ultrasound), and codelivery of other anticancer therapeutic agents should be further explored to increase the response, differentiate the tumor growth rate, and promote tumor regression or indolent growth. For all treatment groups, a reduced concentration of aPD-1 was observed at the terminal time point in comparison to the 3-day time point, indicating drug depletion and suggesting the need for additional interventions to lengthen the presence of the drug within the tumor. Surprisingly, no statistically significant differences were observed among treatment groups, which could be assignable to sample-to-sample variation. However, GG aPD-1 displayed an increase of 1.6- and 3.8-fold of the intratumoral concentration of aPD-1 (μg/mL) as compared to i.p. and intratumoral delivery, respectively, along with a 1.7-fold increase in plasma. This confirms that the GG hydrogel presented herein can serve as an alternative vehicle to deliver, retain, and release aPD-1 in a CRC tumor model.

As research on localized delivery of immunotherapy continues to expand, it is important to mention that it is frequently administered with complex multipolymer systems along with other drugs, in contrast to the single polymer system presented herein. For example, Wang et al. used microneedles in the treatment of melanoma, delivering aPD-1 in combination with dextran nanoparticles and glucose oxidase.^97^ Other options for combination systems include dendritic cell vaccine self-assemble peptide nanofibers with aPD-1, oncolytic viruses, nanocarrier systems, or phototherapy.^98–101^ Wang et al. used microneedles in the treatment of melanoma, delivering aPD-1 in combination with dextran nanoparticles and glucose oxidase.^97^ Other options for combination systems include dendritic cell vaccine self-assemble peptide nanofibers with aPD-1, oncolytic viruses, nanocarrier systems, or phototherapy.^98–101^ As research on localized delivery of immunotherapy continues to expand, it is important to mention that it is frequently administered with complex multipolymer systems along with other drugs, in contrast to the single polymer system presented herein. For example, Wang et al. used microneedles in the treatment of melanoma, delivering aPD-1 in combination with dextran nanoparticles and glucose oxidase.^97^ Other options for combination systems include dendritic cell vaccine self-assemble peptide nanofibers with aPD-1, oncolytic viruses, nanocarrier systems, or phototherapy.^98–101^ Microneedles were used in the treatment of melanoma, delivering aPD-1 in combination with dextran nanoparticles and glucose oxidase.^97^ Other options for combination systems include dendritic cell vaccine self-assemble peptide nanofibers with aPD-1, oncolytic viruses, nanocarrier systems, or phototherapy.^98–101^ Therefore, evaluations with other polymers, alternative form factors, or combination therapies for the delivery of aPD-1 with GG hydrogels (such as chemotherapy or other checkpoint inhibitors) should be further explored.

## Conclusions

This research demonstrates that GG hydrogels are noncytotoxic and can be used as a potential localized delivery system for aPD-1. Challenges such as burst release, drug retention, and large variability require further investigation. Delivery and diffusion of molecules with varying molecular weights and shapes achieved a 70–90% recovery via the disk FRAP method, which confirmed the entrapment phenomena observed in the drug release studies. For the *in vivo* evaluations, while there were no statistically significant differences between the treatment groups that received aPD-1, an increase of 1.5- and 3-fold in the plasma and tumor was observed when delivered intratumorally with the hydrogel. Data suggest that such an approach is comparable and safe as compared to drug-free intratumoral or intraperitoneal injections. In summary, GG hydrogels enable the delivery, release, and retention of aPD-1 intratumorally, which has been shown to be an alternative delivery solution that did not present to be worse, but rather to be equivalent to systemic delivery or bolus injection intratumorally. This delivery method could serve as an adjuvant treatment option, wherein the hydrogel loaded with aPD-1 can be applied in the tumor bed postresection to reduce recurrence. Alternatively, for patients that are not candidates for surgical resection of the tumor, the hydrogel loaded with aPD-1 could be administered intratumorally to enhance the localized presence and manage tumor progression, such that a patient can become eligible for surgical removal. Variations regarding tumor microenvironments and differences between subcutaneous versus orthotopic tumors should be considered in future investigations. This hydrogel presents an opportunity to serve as a platform technology that can be used with other immunotherapy drugs or cancer types.

## Supplementary Material



## Notes

Additional experimental methods for the indirect ELISA protocol developed to quantify aPD-1 and test result graphs for the hydrogel mechanical characterization and long-term *in vitro* aPD-1 release (PDF)
