# Supplementary material for: Evaluation of Trilysine-Cross-Linked Gellan Gum for Intratumoral Delivery of Anti-PD‑1 in a Colorectal Cancer Mouse Tumor Model
Source: ACS Biomater Sci Eng. 2026 Jan 23;12(2):955–70. doi: 10.1021/acsbiomaterials.5c01503 (PMC12892250; doi:10.1021/acsbiomaterials.5c01503)
Supplement: Supplementary file 1 [file ab5c01503_si_001.pdf]

SUPPORTING INFORMATION

Evaluation of Trilysine-Cross-Linked Gellan Gum for Intratumoral Delivery of Anti-PD-1 in a Colorectal Cancer Mouse Tumor Model

Carolina Villarreal-Otalvaro[1,2], Francini Luna[2], Rosangel A. Ramos Espinoza[2], Eric D. Lombardini[2], Zephyr Paxton[1], Luis Vidali[3,4], Jeannine M. Coburn[1,*]

[1]Department of Biomedical Engineering, Worcester Polytechnic Institute, Worcester, MA, US 01609

[2]Boston Scientific Corporation, Marlborough, MA, US 01752

[3]Department of Biology and Biotechnology, Worcester Polytechnic Institute, Worcester, MA, US 01609

[4]Bioinformatics and Computational Biology Program, Worcester Polytechnic Institute, Worcester, MA, US 01609

Total pages: 5

Figure: 4

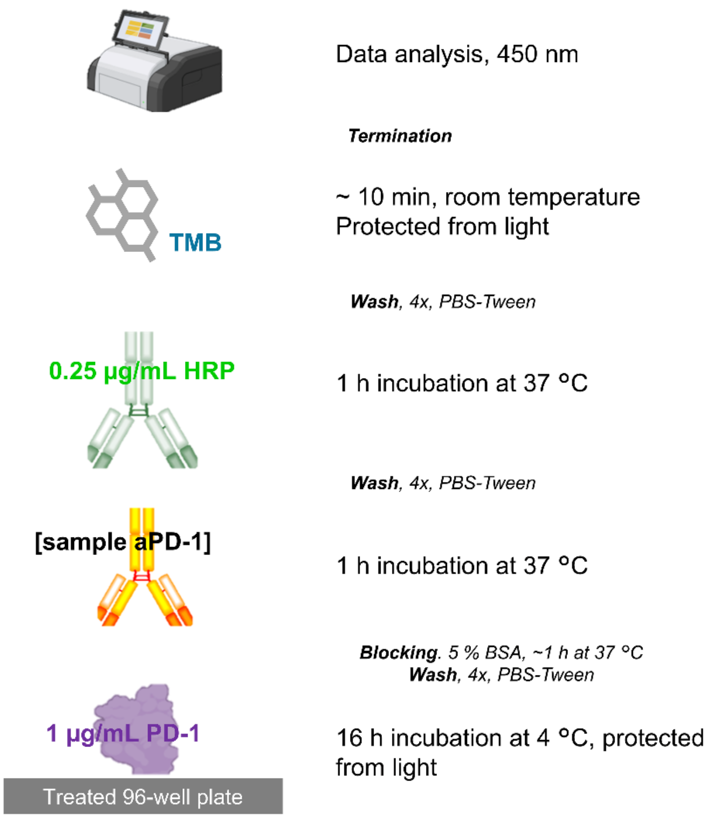

Figure Error! No text of specified style in document.**S1. Indirect ELISA for aPD-1 quantification.** Protocol for aPD-1 concentration from in vitro (hydrogel released) and in vivo (from plasma and tumor samples) evaluations.

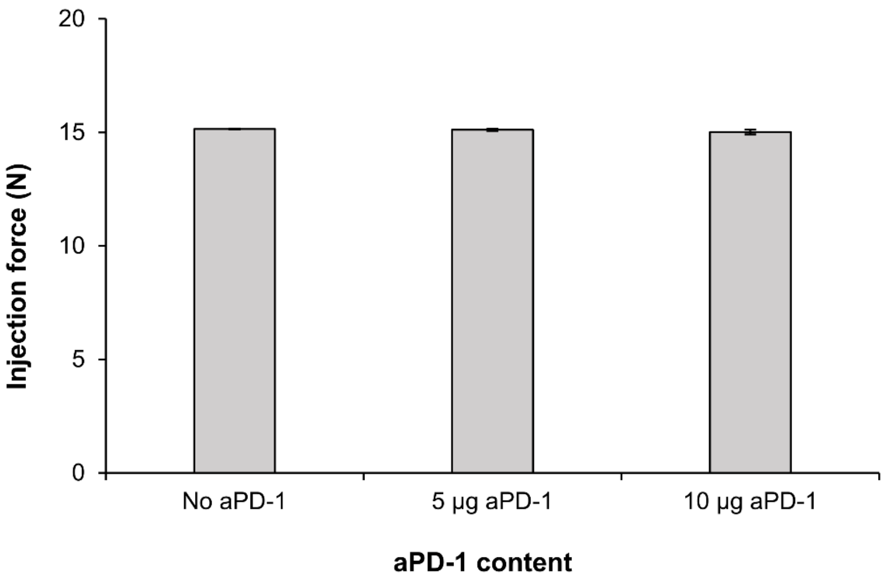

Figure S2. Injection force of the linear plateau region of the GG-based hydrogels formulated with 1% GG and 0.04 trilysine with varying concentrations of aPD-1. Hydrogels were contained in a 1 mL syringe with a 22 Ga needle. All data presented as mean ± std deviation with statistical differences determined by a one-way ANOVA followed by Tukey's HSD test, with a 95% confidence level and $p < 0.05$. Data presented as mean ± std deviation of three replicas. No statistical significance among groups when compared to no aPD-1.

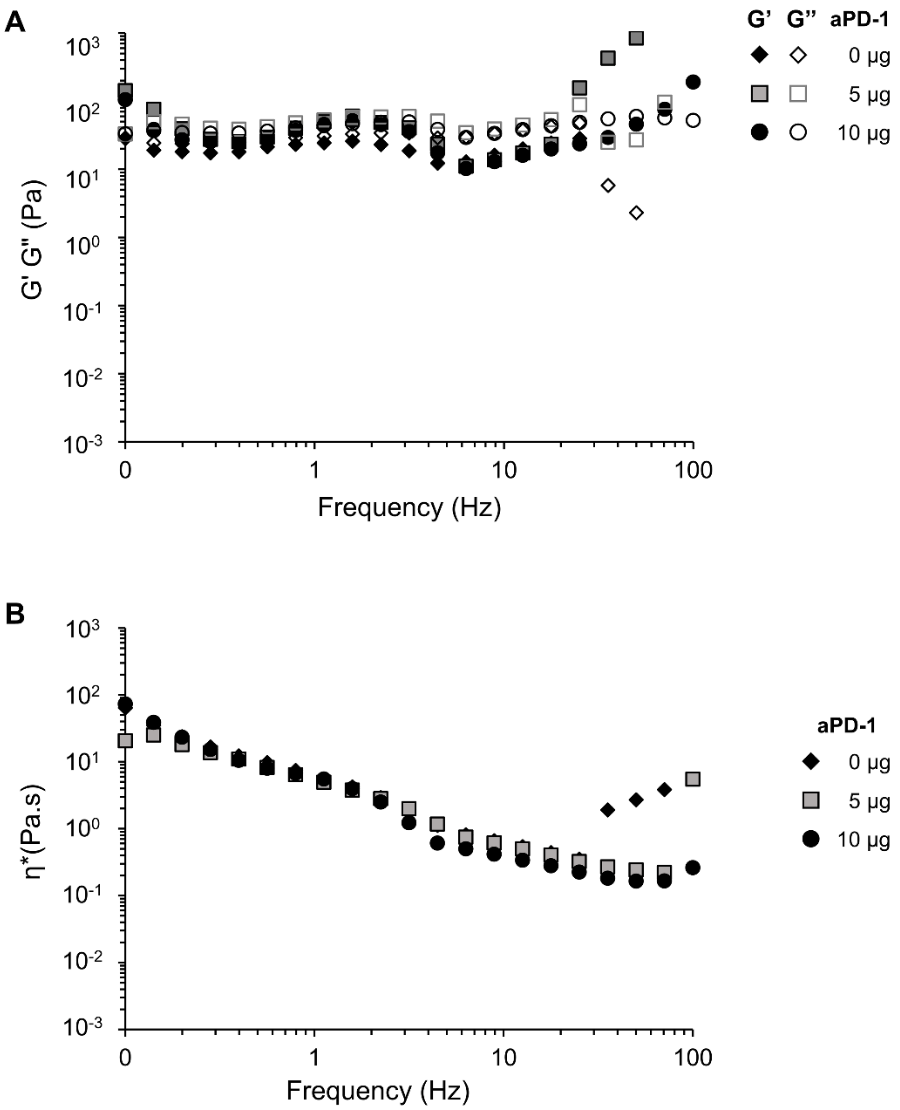

Figure S3. Rheological behavior via frequency sweeps. (**A**) Storage (G’), loss modulus (G”) and (**B**) complex viscosity of 1.0% GG hydrogels crosslinked with 0.04% trilysine with varying concentrations of aPD-1.

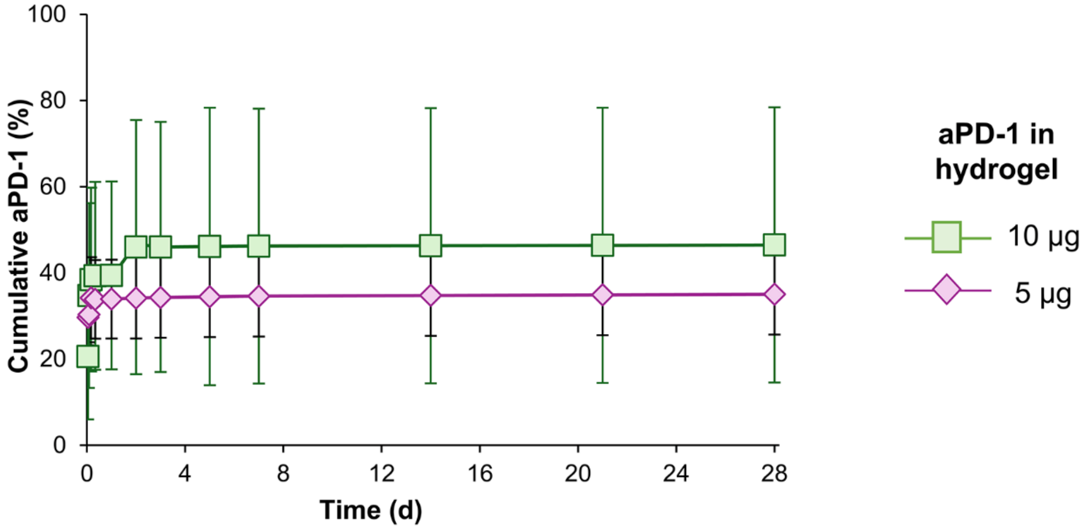

Figure S4. Cumulative release of aPD-1 from GG hydrogels for 28 days. Samples were loaded with 10 μg and 5 μg aPD-1. Representative data shown from three independent samples.
